# Repetitive transcranial magnetic stimulation promotes the recovery of upper limb motor dysfunction in ischemic stroke patients: a DTI-based glymphatic system imaging prospective study

**DOI:** 10.7717/peerj.20709

**Published:** 2026-02-03

**Authors:** Yulan Dong, Jing Liu, Tianyao Wang, Zhaoxiang Zhang, Xiaoyue Hu, Chengjia Zhu, Meijuan Gao, Ping Yan, Hao Lei, Jun Zhou

**Affiliations:** 1 Department of Radiology, The First Affiliated Hospital, Hengyang Medical School, University of South China, Hengyang, Hunan, China; 2 Rehabilitation Medicine Center, The First Affiliated Hospital, Hengyang Medical School, University of South China, Hengyang, Hunan, China; 3 Department of Radiology, Renji Hospital, Shanghai Jiao Tong University School of Medicine, Shanghai, China; 4 Department of Radiology, Changsha Hospital of Traditional Chinese Medicine (Changsha Eighth Hospital), Changsha, Hunan, China

**Keywords:** Glymphatic system, Ischemic stroke, Repetitive transcranial magnetic stimulation, Diffusion tensor image analysis along the perivascular space, Upper limb motor dysfunction

## Abstract

**Background:**

Repetitive transcranial magnetic stimulation (rTMS) is an effective tool for motor function recovery in patients with ischemic stroke (IS). Dysfunction of the glymphatic system is implicated in the pathological process of IS. However, it is still unclear whether the recovery of upper limb motor function in IS is affected by rTMS-driven modulation of glymphatic system function. This study aimed to investigate the potential utility of diffusion tensor imaging analysis along the perivascular space (DTI-ALPS) in assessing the impact of rTMS on IS recovery.

**Methods:**

This prospective study recruited IS participants with upper limb motor disorders. A total of 35 participants were randomly assigned to receive either low-frequency rTMS (LF-rTMS) or high-frequency rTMS (HF-rTMS) for two weeks. HF-rTMS was conducted on the infarcted hemisphere M1 and the LF-rTMS was conducted on the non-infarcted hemisphere M1. The information collected from each patient included clinical characteristics, upper limb motor function score, and bilateral hemisphere DTI-ALPS index. Paired *t*-tests were used to evaluate longitudinal changes of the DTI-ALPS index in LF-rTMS and HF-rTMS groups. The DTI-ALPS index change rates were calculated, and their differences were compared between groups. Spearman’s correlation analyses were performed to assess the relationships between DTI-ALPS index changes and changes in upper limb motor function score.

**Results:**

At baseline, no significant differences were observed in the DTI-ALPS index between the HF-rTMS and LF-rTMS groups. After two weeks of rTMS treatment, the DTI-ALPS index of the non-stimulated hemisphere in the HF-rTMS group significantly decreased (*t* = 2.42, *P* = 0.028), and the change rate of the DTI-ALPS index was negatively correlated with the recovery of upper limb motor function (*r* =  − 0.42, *P* = 0.011). In the LF-rTMS group, longitudinal analysis showed improvement in upper limb motor function scores, but there was no significant change in the DTI-ALPS index.

**Conclusion:**

Both HF-rTMS and LF-rTMS are beneficial for the rehabilitation of upper limb motor function in patients with IS, but the different changes in DTI-ALPS index may indicate differential effects of HF-rTMS and LF-rTMS on the glymphatic system. However, more research is needed to confirm the underlying mechanisms of this interaction.

## Introduction

Ischemic stroke (IS), the most prevalent form of stroke, is a leading cause of disability globally, with its incidence continuing to rise annually (Campbell et al., 2019; Powers, 2020; Feigin et al., 2021). A significant proportion of ischemic stroke patients experience motor dysfunction, particularly affecting the upper limb (UL; Xu et al., 2021). The motor function of the upper limb is complex and plays a vital role in everyday activities. When recovery is suboptimal, survivors can face long-term disability, significantly diminishing their quality of life and placing a heavy burden on their families. In contrast to lower limb dysfunction, recovery of upper limb motor function is typically slower, and fine motor recovery often takes an extended period with outcomes that are not always satisfactory (Pollock et al., 2014). Consequently, both patients and their families may lose confidence in the rehabilitation process.

Repetitive transcranial magnetic stimulation (rTMS) is a non-invasive neurostimulation technique that has been shown to provide positive effects for ischemic stroke patients. These benefits include improvements in cortical excitability, promotion of neurogenesis, enhancement of neurological function post-stroke, increased angiogenesis around the infarct area, and control of inflammation (Zong et al., 2022; Zhou et al., 2023). As a result, rTMS has emerged as a promising therapeutic approach in stroke rehabilitation (Zong et al., 2022; Luo et al., 2022; Kemps et al., 2022). The 2019 stroke treatment guidelines recommend rTMS for treating subacute stroke upper limb motor dysfunction (Level A; Lefaucheur et al., 2020). Research indicates that rTMS can modulate microenvironment factors at the injury site, regulate neuroglial cell activity, and repair nerve fiber myelin, thereby promoting the reconstruction of synapses and neural circuits (Caglayan et al., 2019; Li et al., 2020). However, a significant number of patients still fail to fully recover and are left with varying degrees of functional impairment (Raghavan, 2015). Therefore, while ongoing advancements in rTMS technology are essential, it is equally important to explore new target sites and potential therapeutic mechanisms for rTMS.

The glymphatic system pathway plays a crucial role in the clearance of brain waste by facilitating the movement of cerebrospinal fluid (CSF) through arterial perivascular spaces into the brain. This process occurs *via* aquaporin 4 (AQP4) water channels located in perivascular astrocytes, followed by the promotion of outflow through venous perivascular spaces, effectively clearing waste from the brain (Rasmussen, Mestre & Nedergaard, 2018). Inflammation can lead to the loss of AQP4 polarity, reducing the efficiency of waste clearance; however, rTMS may enhance the glymphatic system by improving the polarity of AQP4 (Wu et al., 2022; Cai et al., 2024). In a mouse model of Alzheimer’s disease, rTMS treatment can enhance the exchange efficiency of CSF-ISF in facilitating waste clearance, while also inhibiting the activation of microglia and astrocytes, thereby reducing the release of pro-inflammatory cytokines (Lin et al., 2021; Wu et al., 2022). rTMS treatment can improve the glymphatic system in intracerebral hemorrhage mice, enhancing the clearance of RITC-dextran and FITC-dextran in brain parenchyma and improving neurological functions (Liu et al., 2023). Following ischemic stroke, research suggests that dysfunction in the glymphatic system impairs the clearance of inflammatory mediators and leads to the accumulation of neurotoxic metabolites (Passarelli, Nimjee & Townsend, 2024). This disruption can hinder motor function recovery by obstructing the brain’s normal waste removal processes and tissue repair mechanisms (Wang et al., 2023). Additionally, after ischemic stroke, an increase in CSF transport into the brain parenchyma, combined with the blockage of interstitial fluid (ISF) outflow, can result in the abnormal accumulation of cerebral tissue fluid. This may lead to brain edema, which can cause long-term functional deficits and delay neurological recovery (Zhu et al., 2024). Therefore, further research is needed to assess the relationship between glymphatic system remodeling in ischemic stroke patients and the effects of rTMS treatment.

Diffusion magnetic resonance imaging has emerged as a valuable non-invasive tool for assessing the glymphatic system, which plays a crucial role in the clearance of metabolic waste from the brain. Diffusion tensor imaging analysis along the perivascular space (DTI-ALPS) is a sophisticated non-invasive method based on diffusion tensor imaging that allows for the evaluation of the human glymphatic system, providing insights into the dynamics of cerebrospinal fluid flow and interstitial fluid drainage (Taoka et al., 2017; Taoka et al., 2024). DTI-ALPS index estimates the diffusion rate of medullary veins and the space around the vessels and has been applied in clinical settings to assess glymphatic system activity in various conditions, including Alzheimer’s disease (Taoka et al., 2017; Steward et al., 2021), normal pressure hydrocephalus (Bae et al., 2021), Parkinson’s disease (McKnight et al., 2021), age-related iron deposition (Zhou et al., 2020), diabetes-related cognitive impairment (Yang et al., 2020), brain edema associated with tumors (Toh, Siow & Castillo, 2021), obesity (Yi et al., 2025), and ischemic stroke (Toh, Siow & Castillo, 2021; Qin et al., 2023).

In summary, DTI-ALPS may serve as a quantitative imaging biomarker for monitoring the brain’s ability to clear metabolic waste (Wei et al., 2023; Huang et al., 2024). However, there is a lack of research investigating the relationship between ischemic stroke and rTMS using DTI-ALPS. Therefore, the purpose of this study is to use DTI-ALPS index to assess the glymphatic system in patients with ischemic stroke, to identify changes in DTI-ALPS index before and after rTMS treatment, and to identify factors linked to these changes. This will provide objective evidence for the therapeutic mechanisms of rTMS.

## Materials and Methods

### Participants and clinical information

The research protocol was endorsed by the Medical Ethics Committee of the First Affiliated Hospital of the University of South China (approval number 2021KS-KF-13-01). Participants were enrolled continuously in a single-center, prospective study conducted from January 2022 to June 2023. The inclusion criteria were as follows: (1) Initial stroke episode, consistent with the 2021 American Heart Association/American Stroke Association diagnostic criteria for stroke (Kleindorfer et al., 2021). (2) Unilateral presentation characterized by persistent upper limb motor impairment. Participants must exhibit hand motor function within Brunstrom stages II to V or a modified Ashworth scale score not exceeding Grade III. It was required that upper limb motor function was normal prior to the stroke. (3) Disease duration of less than six months post-stroke. (4) Right-handed and age range of 18 to 80 years. Exclusion criteria included: (1) Bilateral cerebral infarction. (2) Contraindications to MRI. (3) Severe neurological or psychiatric conditions such as Alzheimer’s disease, Parkinson’s disease, schizophrenia, or epilepsy. (4) Inability to calculate the DTI-ALPS index due to excessive head movement during scanning. (5) Abnormal blood lymphocyte counts. The neurologist documented the estimated onset time of each participant’s stroke. All subjects underwent neuropsychological assessments and multiparametric MRI scans. All participants voluntarily joined the study and gave their written informed consent.

**Figure 1:**
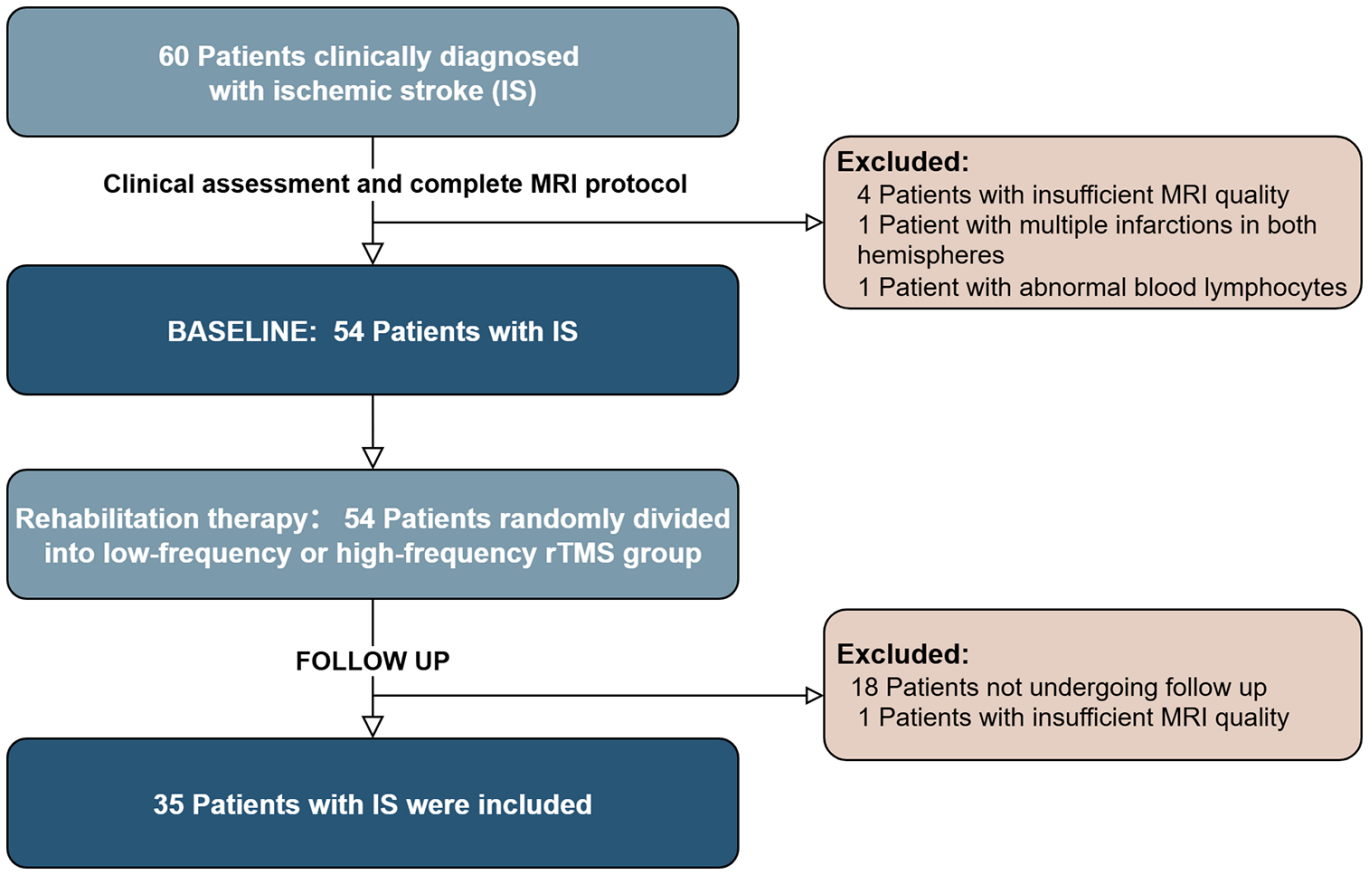
Flowchart shows the inclusion and exclusion criteria of the study.

Sixty participants underwent neuropsychological testing and MRI scans. During the baseline period, six patients were excluded due to severe head motion during MR scanning (*n* = 4), bilateral cerebral infarction (*n* = 1), and abnormally increased lymphocyte count (*n* = 1). Follow up assessment included the same clinical scales and neuroimaging measures as the baseline period. Nineteen patients were excluded due to requested termination of the trial (*n* = 18) and severe head motion during MR scanning (*n* = 1). The final 35 subjects were included in the final statistical analysis (Fig. 1). All evaluations were completed before the MRI examination and performed by a rehabilitation physician one-on-one with the participants.

### MR imaging acquisition

All subjects were examined with a 3.0 T MRI clinical scanner (MAGNETOM Prisma, Siemens Healthcare, Erlangen, Germany) using a Nova 64-channel brain phased array coil. Diffusion tensor imaging was performed using a single-shot echo-planar imaging (EPI) with the following parameters: TR/TE = 5,800 ms/83 ms; diffusion gradient encoding in 20 directions; *b* = 0, 1,000 s/mm^2^. Conventional MRI sequences included 3D-T1 (repetition time = 7.3 ms, echo time = 3 ms, flip angle = 15°, slice thickness = one mm without slice gap, matrix = 256 × 256, field of view = 240 mm), T2-FLAIR (repetition time = 8,000 ms, echo time = 150 ms, inversion time = 2,100 ms, flip angle = 90°, slice thickness = four mm without slice gap, matrix = 256 × 256, field of view = 24 cm), and DWI (repetition time = 5,000 ms, echo time = 86 ms, and b value = 1,000 s/mm^2^, along three orthogonal directions, flip angle = 90°, slice thickness = four mm, matrix = 256 × 256, and field of view = 240 mm).

### Calculation of the DTI-ALPS index

Non-invasive diffusion tensor imaging was used to assess the along the perivascular space at the level of the lateral ventricle body, reflecting the glymphatic system in stroke patients. At the level of the lateral ventricular body, medullary veins and its perivascular spaces extended in the left–right (*x*-axis) direction of the image coordinate. The projection fibers are adjacent to the lateral ventricular wall, extending in the up-down direction (*i.e.,* the *z*-axis direction of the image coordinate). The association fibers (superior longitudinal fasciculus) are located on the outside of the projection fibers, extending in the anterior-posterior direction (*i.e.,* the *y*-axis direction of the image coordinate). The perivascular space is perpendicular to the projection fibers and association fibers. In the *x*-axis direction, the *x*-axis diffusion characteristics of the projection fibers and association fibers are represented by Dxx_proj_ and Dxx_associ,_ respectively. In the *y*-axis direction, the diffusion characteristics of the projection fibers are represented by Dyy_proj_. In the *z*-axis direction, the diffusion characteristics of the association fibers are represented by Dzz_associ_. To quantify cerebral glymphatic system activity, the DTI-ALPS index is defined as follows:

ALPS index = $ \frac{\mathrm{mean} \left( \mathrm{Dxxproj},\mathrm{Dxxassoci} \right) }{\mathrm{mean} \left( \mathrm{Dyyproj},\mathrm{Dzzassoci} \right) } $ (Taoka et al., 2017)

**Figure 2:**
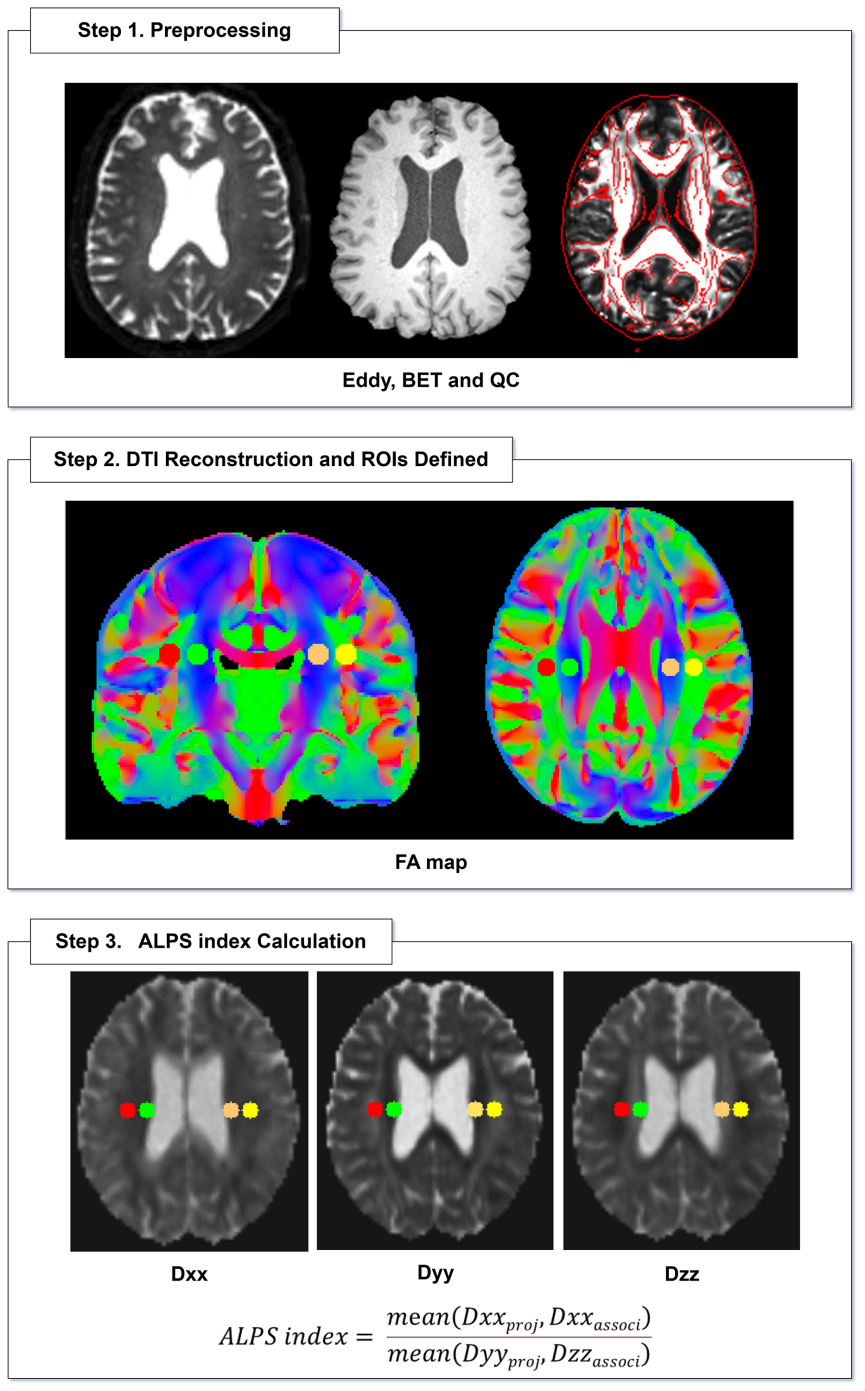
Flowcharts for DTI-ALPS processing. Directionally encoded color FA map illustrates ROIs of projection (blue area) and association (green area) fibers in bilateral periventricular regions. BET, brain extraction tool; QC, quality control; FA, fractional anisotropy; Dxx, diffusivity along the *x*-axis; Dyy, diffusivity along the *y*-axis; Dzz, diffusivity along the *z*-axis; ALPS, diffusion tensor imaging analysis along the perivascular space.

The DTI parameters were calculated using the FMRIB Software Library (FSL) toolbox (version 5.0.1; https://fsl.fmrib.ox.ac.uk/fsl/fslwiki). For each subject, color fractional anisotropy (FA) mapping and the diffusivity values in the directions of the *x*-axis (Dxx), *y*-axis (Dyy), and *z*-axis (Dzz) were generated. A circular region of interest (ROI) with a diameter of five mm was consistently delineated at the level of the lateral ventricles in the projection fibers area and the associated fibers area of the color FA mapping. The researchers avoided drawing ROIs on brain lesions such as lacunar lesions or white matter hyperintensities. The DTI-ALPS index of each subject was calculated to reflect their individual glymphatic system activity. An example of ROI placement for DTI-ALPS index measurement is shown in (Fig. 2).

### rTMS

**Figure 3:**
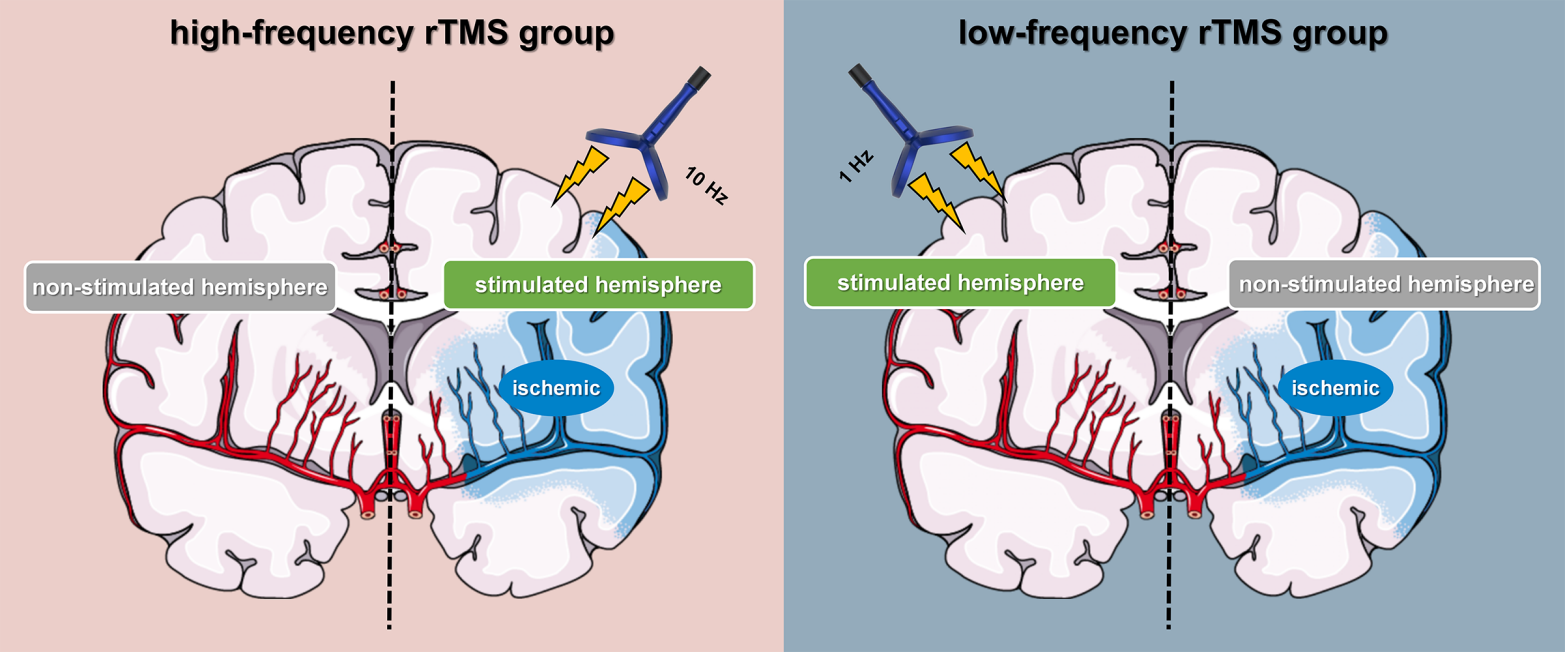
Schematic diagram of high-frequency and low-frequency rTMS treatment. HF-rTMS is used to reactivate cortical excitability in the infarct side (the infarct side belongs to the stimulated hemisphere). LF-rTMS is used to reduce cortical excitability in the intact side (the intact side belongs to the stimulated hemisphere).

All subjects underwent two weeks of repetitive transcranial magnetic stimulation therapy using an 8-shaped coil system manufactured by YIRUIDE (Wuhan, China). All subjects were randomly assigned to the high-frequency rTMS group or the low-frequency rTMS group. The subjects and rTMS operators were blinded and unaware of the grouping information. The high-frequency rTMS group received 10 Hz rTMS (100% rMT, 10 Hz stimulation, 1,200 pulses) treatment for the infarcted hemisphere’s primary motor cortex (M1) area. In contrast, the low-frequency rTMS group received 1 Hz rTMS (100% rMT, 1 Hz, 1,200 pulses) treatment for the M1 area of the non-infarcted hemisphere motor cortex (Fig. 3). During rTMS treatment, the subject was instructed to keep still, minimize head movement, and use earplugs to reduce noise stimulation. The intersection of the figure-eight coil was positioned tangentially to the scalp over M1 of the stimulated hemisphere, and the coil tilt was adjusted to 30°–45° relative to the mid-sagittal plane.

### Statistics

Statistical analysis was performed with SPSS 25 (IBM, Armonk, NY) and R v4.1.2 (R Core Team, 2021). The Shapiro–Wilk test was used to evaluate the normality of the data distribution. Demographic data were compared by using Chi-squared tests for nominal variables and Student’s *t*-tests or Mann–Whitney *U* tests for continuous variables. The difference in DTI-ALPS index longitudinal changes between the LF-rTMS and HF-rTMS groups was evaluated using paired *t*-tests. The difference in upper limb motor function score changes between the LF-rTMS and HF-rTMS groups was evaluated using a Wilcoxon signed rank test. The change rate of the DTI-ALPS index was calculated as follows: $ \frac{ \frac{\mathrm{FU}-\mathrm{BL}}{\mathrm{BL}} }{\mathrm{FU}-\mathrm{time}(\mathrm{days})} \ast 100\%$. To investigate the differences in the DTI-ALPS index or change rate of the DTI-ALPS index between groups, this study first applied the two-sample *t*-test followed by a multivariable linear regression analysis adjusted for age, sex, BMI, and time since stroke onset. The correlations between the DTI-ALPS index changes and changes in upper limb motor function score were evaluated by using Spearman’s correlations and multivariable linear regression models adjusted for age, sex, BMI, and time since stroke onset. *P* < 0.05 was considered to indicate statistical significance, and *P* values were corrected for multiple comparisons with the false discovery rate.

## Results

### Participant characteristics

The final group of subjects comprised 35 ischemic stroke (IS) patients, including 18 in the LF-rTMS group (13 males; average age 57.2 ± 6.2 years; age range 47–69 years) and 17 in the HF-rTMS group (11 males; average age 60.8 ± 6.5 years; age range 50–74 years). The two groups were divided into age (*t* = −1.65, *P* = 0.14) and gender (*χ*^2^ = 0.23, *P* = 0.632) aspect matching. Table 1 summarizes the demographic and clinical characteristics of the subjects.

**Table 1:** Demographics and clinical characteristics of the participants. Except where indicated, data are numbers of participants. Student *t*-test and Mann-Whitney *U* test were used for continuous variables, and Pearson *χ*^2^ test was used for nominal variables.

	high-frequency rTMS group	low-frequency rTMS group	*χ*^2^/t/Z	*P*
Number of cases	17	18		
Age (years)^a^	60.8 ± 6.5	57.2 ± 6.2	1.65	0.108
Sex (male/female)	11/6	13/5	0.23	0.632
BMI (kg/m^2^)^a^	23.0 ± 3.9	24.8 ± 3.0	−1.56	0.128
Lymphocyte counts (×10^9^/L)^a^	1.6 ± 0.5	1.6 ± 0.6	0.01	0.996
NIHSS^a^	7.6 ± 2.6	7.8 ± 2.8	−0.27	0.792
Time since stroke onset (day)^b^	10 (6)	12.5 (48)	−0.55	0.585
Brunstrom^b^	2.0 (2.0)	3.0 (1.0)	−0.70	0.483
Fugl-Meyer Assessment^b^	7.0 (9.0)	7.5 (10.0)	−0.12	0.908
Motor assessment scale	0 (4)	0 (1)	−0.51	0.612
Modified ashworth scale	0 (1)	1 (2)	−1.49	0.173
SWMT	2 (2)	2 (2)	−0.58	0.590
Wolf Motor Function Test^b^	1.0 (12.0)	5.0 (11.0)	−0.23	0.821
ADL^a^	55.7 ± 22.6	50.3 ± 16.7	0.81	0.423
QOL^b^	160.0 (39.0)	148.5 (38.0)	−1.00	0.314

**Notes.**

a Data are mean ± standard deviation (SD).

b Data are median, and data in parentheses are the interquartile range.

BMI body mass index NIHSS National Institutes of Health Stroke Scale ADL activities of daily living QOL quality of life SWMT Semmes-Weinstein Monofilament Test

### Comparison of the DTI-ALPS index between the HF-rTMS group and the LF-rTMS group

Table 2 shows the comparison of the DTI-ALPS index for the total cerebral, stimulated hemisphere, and non-stimulated hemisphere between the HF-rTMS group and the LF-rTMS group.

**Table 2:** DTI-ALPS index in different brain regions in study participants.

Region		DTI-ALPS index			Multiple linear regression		
		High-frequency rTMS group	Low-frequency rTMS group	Uncorrected *P* value^a^	*β*	95% CI	*P*value^b^
Total cerebral	BL	1.222 ± 0.105	1.213 ± 0.103	0.795	0.013	−0.061, 0.087	0.725
	FU	1.212 ± 0.099	1.221 ± 0.104	0.808	0.029	−0.043, 0.102	0.419
Stimulated hemisphere	BL	1.235 ± 0.117	1.235 ± 0.108	0.997	0.015	−0.068, 0.098	0.711
	FU	1.233 ± 0.110	1.244 ± 0.107	0.761	0.024	−0.054, 0.103	0.535
Non-stimulated hemisphere	BL	1.209 ± 0.102	1.190 ± 0.106	0.603	0.011	−0.062, 0.084	0.765
	FU	1.192 ± 0.097	1.198 ± 0.108	0.873	0.034	−0.038, 0.106	0.343

**Notes.**

Data are presented as mean ± standard deviation (SD).

a Computed by using Student *t*-test.

b Compared using a multiple linear regression model and adjusted for age, sex, BMI, and time since stroke onset.

BL baseline FU follow up CI confidence interval

At baseline, the DTI-ALPS index of the LF-rTMS group was 1.213 ± 0.103 (total cerebral), 1.235 ± 0.108 (stimulated hemisphere), and 1.190 ± 0.106 (non-stimulated hemisphere). The DTI-ALPS index of the HF-rTMS group was 1.222 ± 0.105 (total cerebral), 1.235 ± 0.117 (stimulated hemisphere), and 1.209 ± 0.102 (non-stimulated hemisphere). The two groups had no significant difference in the DTI-ALPS index of the total cerebral, stimulated, or non-stimulated hemisphere, even adjusting for age, gender, BMI, and time since stroke onset.

During the follow-up, the DTI-ALPS index of the LF-rTMS group was 1.221 ± 0.104 (total cerebral), 1.244 ± 0.107 (stimulated hemisphere), and 1.198 ± 0.108 (non-stimulated hemisphere). The DTI-ALPS index of the HF-rTMS group was 1.212 ± 0.099 (total cerebral), 1.233 ± 0.110 (stimulated hemisphere), and 1.192 ± 0.097 (non-stimulated hemisphere). There was no significant difference in the DTI-ALPS index in the total cerebral, stimulated, or non-stimulated hemisphere, even adjusting for age, gender, BMI, and time since stroke onset.

### Longitudinal changes of clinical scale

**Figure 4:**
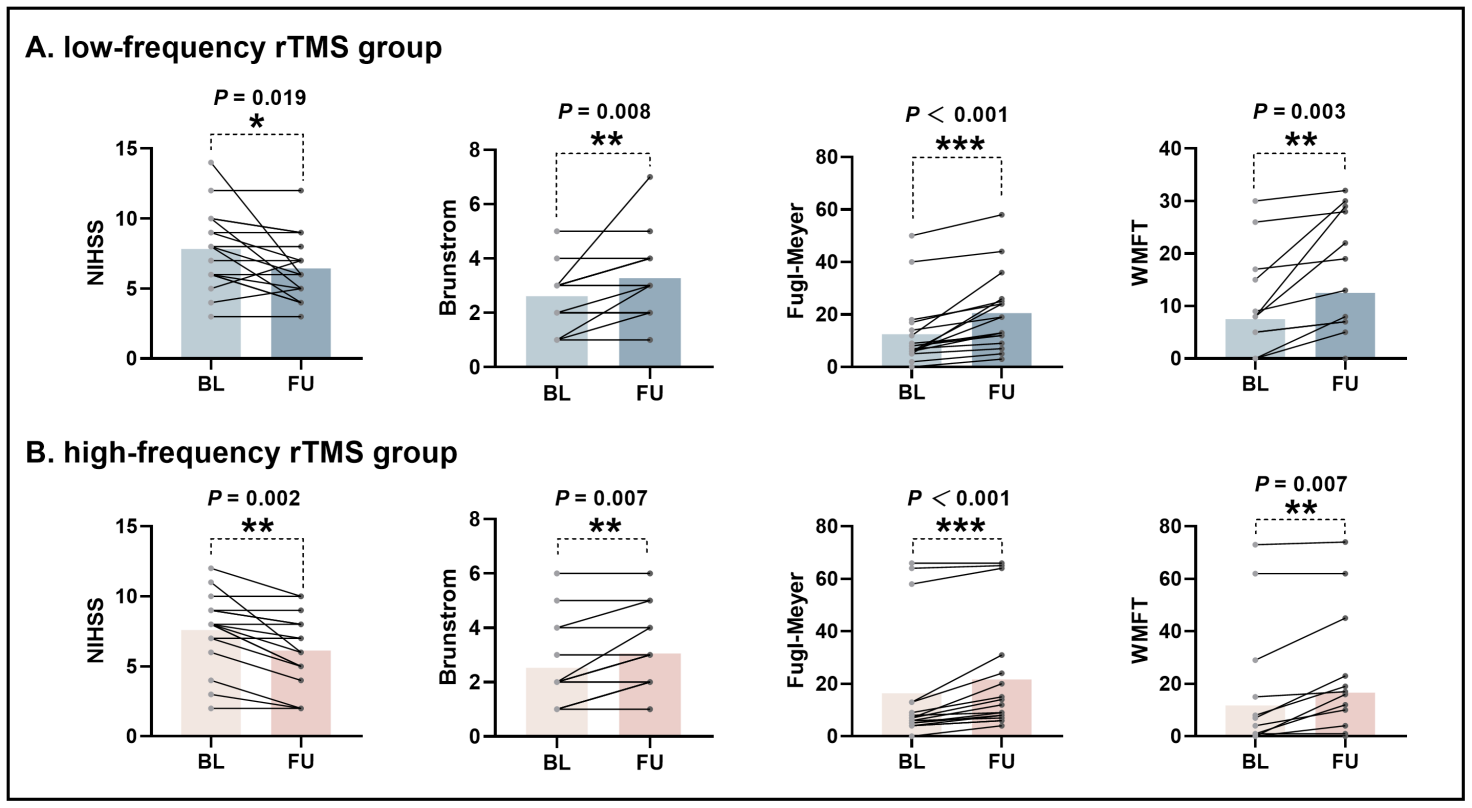
Longitudinal changes in UL motor function of participants in the low-frequency rTMS group and high-frequency rTMS group. Computed by using Wilcoxon signed rank test . * indicates *P* < 0.05; ** indicates *P* < 0.01; *** indicates *P* < 0.001. BL, baseline; FU, follow up; NIHSS, National Institutes of Health Stroke Scale; WMFT, Wolf Motor Function Test.

Figure 4 illustrates longitudinal changes in clinical scale between baseline and follow-up assessments for the HF-rTMS group (Fig. 4A) and LF-rTMS group (Fig. 4B). Compared to baseline, both groups exhibited significant improvements in the National Institutes of Health Stroke Scale (NIHSS), Brunnstrom Stages, Fugl-Meyer Assessment (FMA), and Wolf Motor Function Test (WMFT) scores (*P* < 0.05). In contrast, longitudinal analysis showed no significant changes in the scores of both groups on the Motor Assessment Scale, Modified Ashworth Scale, or Semmes-Weinstein Monofilament Test (SWMT) scores (Table S1).

### Longitudinal changes of DTI-ALPS index

#### Change of the DTI-ALPS index in study participants after rTMS treatment

**Figure 5:**
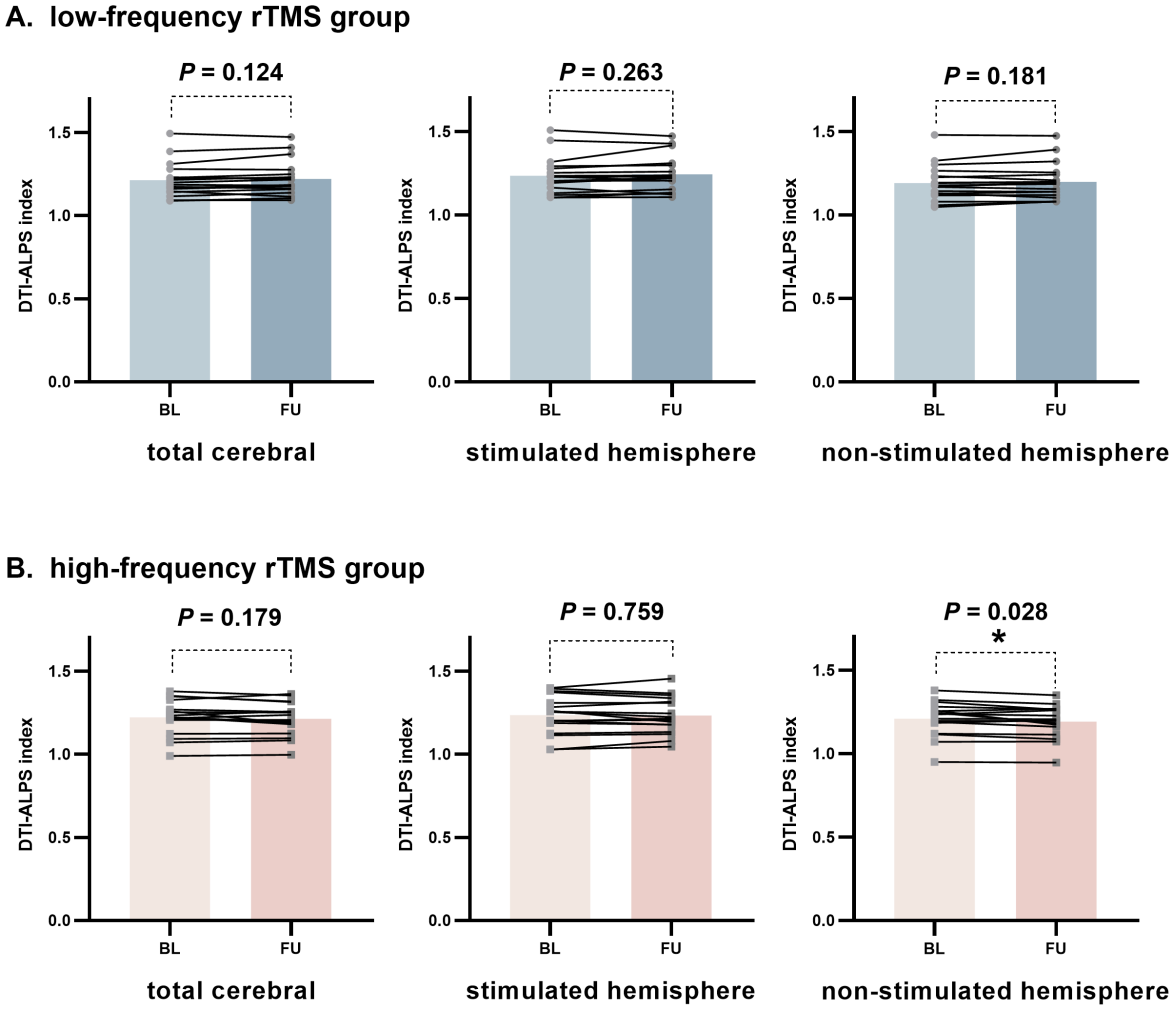
Longitudinal changes in DTI-ALPS index of participants in the low-frequency rTMS group and high-frequency rTMS group. Computed by using paired-samples *t*-test. *indicates *P* < 0.05, **indicates *P* < 0.01, ***indicates *P* < 0.001. BL, baseline; FU, follow up.

Figure 5 shows changes in the DTI-ALPS index between the baseline and follow-up periods in the LF-rTMS group (Fig. 5A) and the HF-rTMS group (Fig. 5B). Compared to the baseline, the DTI-ALPS index in the non-stimulated hemisphere of the HF-rTMS group showed a significant downward trend during the follow-up period (*t* = 2.42, *P* = 0.028). Although the DTI-ALPS index for the total cerebral and stimulated hemisphere in the HF-rTMS group also decreased to some extent during the follow-up, these differences were not statistically significant (DTI-ALPS index in the HF-rTMS group: total cerebral, *t* = 1.41, *P* = 0.179; stimulated hemisphere, *t* = 0.31, *P* = 0.759). In contrast, for the LF-rTMS group, there was no significant statistical difference in the follow-up DTI-ALPS index between the total cerebral, stimulated hemisphere, and non-stimulated hemisphere compared to baseline (total cerebral, *t* = −1.62, *P* = 0.124; stimulated hemisphere, *t* = −1.16, *P* = 0.263; non-stimulated hemisphere, *t* = −1.40, *P* = 0.181).

#### Comparison of the change rate of the DTI-ALPS index between the HF-rTMS group and LF-rTMS group

Table 3 summarizes the change rate of the DTI-ALPS index after treatment with HF-rTMS and LF-rTMS for two-week periods, which was statistically analyzed in the total cerebral, stimulated, and non-stimulated hemispheres. There was a significant statistical difference (HF-rTMS *vs.* LF-rTMS; *t* = −2.61, *P* = 0.013, P_FDR_ = 0.040) in the change rate of the DTI-ALPS index in the non-stimulated hemisphere between the HF-rTMS group and the LF-rTMS group. However, no such effect was observed in the two groups’ total cerebral and stimulated hemisphere (HF-rTMS *vs.* LF-rTMS; total cerebral, *t* = −1.81, *P* = 0.079; stimulated hemisphere, *t* = −0.80, *P* = 0.428).

**Table 3:** Change rate of the DTI-ALPS index in study participants after rTMS treatment.

Change rate of the DTI-ALPS index	high-frequency rTMS group	low-frequency rTMS group	t	Uncorrected *P* value	FDR corrected *P* value
Total cerebral	−0.061 ± 0.169	0.033 ± 0.137	−1.81	0.079	0.114
Stimulated hemisphere	−0.013 ± 0.199	0.041 ± 0.197	−0.80	0.428	0.438
Non-stimulated hemisphere	−0.108 ± 0.178	0.027 ± 0.124	−2.61	0.013^*^	0.040^*^

**Notes.**

Data are presented as mean ± standard deviation (SD). Computed by using Student *t*-test.

* Significant after correction for false discovery rate.

The change rate of DTI-ALPS index was calculated as follows:((FU-BL)/BL)/FU-time (days)*100%.

BL baseline FU follow up FU-time (days) follow-up time

There was no statistically significant difference in the follow-up interval between the two groups 14 ± 3 (days) in the HF-rTMS group and 13 ± 3 (days) in the LF-rTMS group (*t* = 1.18, *P* = 0.248).

#### Associations between the DTI-ALPS index and clinical scale in HF-rTMS group

**Figure 6:**
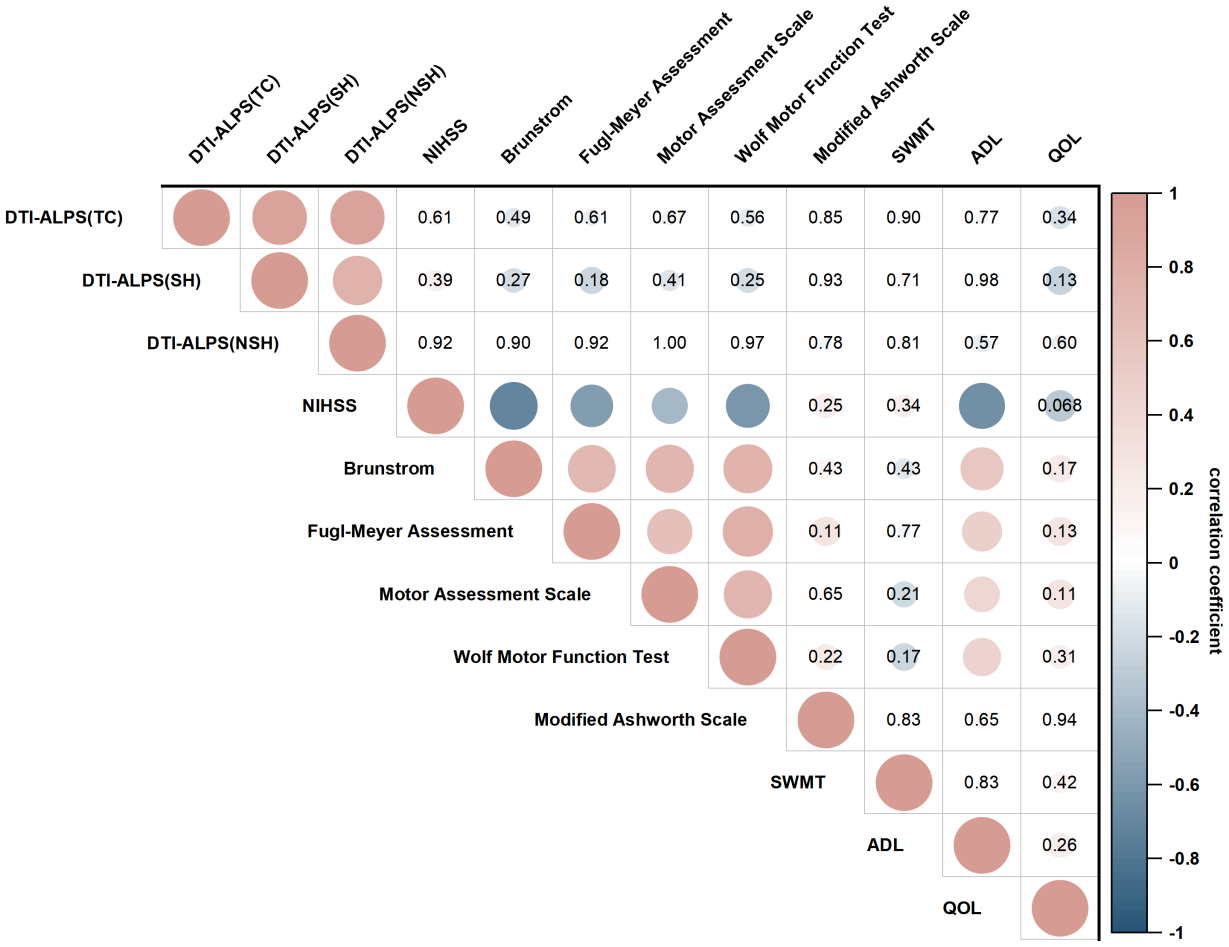
Associations between the DTI-ALPS index and clinical characteristics in baseline. Computed by using Spearman’s correlations and multivariable linear regression models adjusted for age, sex, BMI, and time since stroke onset. Blank indicates significant correlation (*P* < 0.05, non-correction for false discovery rate). TC, total cerebral; SH, stimulated hemisphere; NSH, non- stimulated hemisphere; NIHSS, National Institutes of Health Stroke Scale; SWMT, Semmes-Weinstein Monofilament test; ADL, activities of daily living; QOL, quality of life.

**Figure 7:**
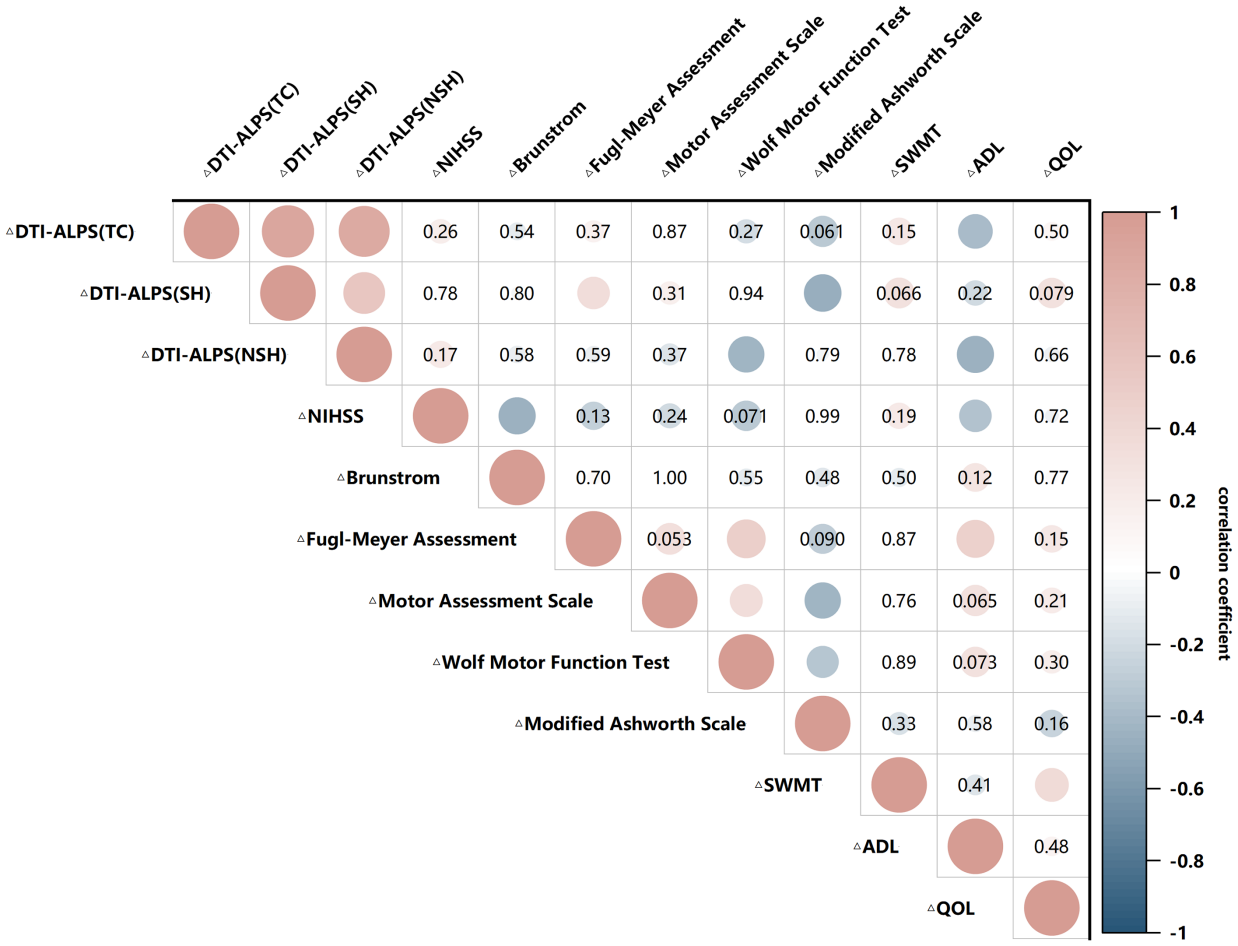
Association between change in DTI-ALPS index and change in UL motor function. Computed by using Spearman’s correlations and multivariable linear regression models adjusted for age, sex, BMI, and time since stroke onset. Blank indicates significant correlation (*P* < 0.05, non-correction for false discovery rate). Delta (Δ) indicates the change. The change in DTI-ALPS index was calculated as follows: ((FU−BL)/BL)/FU− time(days)∗100%. The change in clinical characteristics was calculated as follows: FU –BL. BL, baseline; FU, follow up; FU-time, follow-up time; TC, total cerebral; SH, stimulated hemisphere; NSH, non-stimulated hemisphere; NIHSS, National Institutes of Health Stroke Scale; SWMT, Semmes-Weinstein Monofilament test; ADL, activities of daily living; QOL, quality of life.

In the HF-rTMS group, there were no significant cross-sectional correlations observed between baseline DTI-ALPS index (total cerebral, stimulated hemisphere, and non-stimulated hemisphere) and Wolf Motor Function Test (WMFT) scores (Fig. 6). However, longitudinal analyses demonstrated a negative association between the change rate of DTI-ALPS index in the non-stimulated hemisphere and the change of WMFT (*r* = −0.42, *P* = 0.011, P_FDR_ = 0.102; Fig. 7). Additionally, the change rate of DTI-ALPS index in the stimulated hemisphere showed a positive correlation with the change of Fugl-Meyer Assessment (*r* = 0.35, *P* = 0.042, P_FDR_ = 0.226). No significant associations were detected between total cerebral DTI-ALPS index change and upper limb motor recovery.

## Discussion

This study recruited participants with ischemic stroke and used the DTI-ALPS index to estimate glymphatic function. The association between longitudinal changes in the DTI-ALPS index and upper limb motor function improvement was assessed after participants underwent HF-rTMS or LF-rTMS treatment. The baseline DTI-ALPS index did not show any significant differences between the HF-rTMS and LF-rTMS groups. Similarly, there were no significant differences in baseline upper limb motor function scale scores between the two groups. After two weeks of rehabilitation treatment, both the LF-rTMS and HF-rTMS groups exhibited significant improvements in upper limb function.

Recently, the understanding of the mechanism of post-stroke motor function rehabilitation mainly focuses on the interhemispheric competition model (IHI; Safdar et al., 2023). This model proposes that the infarcted hemisphere faces a dual challenge: primary ischemic damage and excessive inhibitory effects from the intact side. rTMS uses patterned electromagnetic pulses to affect cortical excitability through neuromodulation mechanisms. Empirical evidence demonstrates differential effects based on stimulation frequency: high-frequency protocols produce cortical facilitation, whereas low-frequency stimulation induces cortical inhibition. HF-rTMS is used to reactivate cortical excitability in the infarct side. LF-rTMS is used to suppress excitability in the intact hemisphere, thereby attenuating pathological transcallosal inhibition exerted on the infarcted hemisphere and reinstating balanced interhemispheric inhibition (Di Pino et al., 2014; Sheng et al., 2023). rTMS promotes neurological function recovery after a stroke, and this effect is mediated through multiple pathways (Zong et al., 2022; Zhou et al., 2023). rTMS can enhance neuronal uptake of glutamate and decrease its accumulation between neurons by upregulating glutamate receptor activity or expression, thereby reducing neuronal apoptosis or necrosis caused by excitotoxicity (Poh et al., 2019). It can also induce the polarization of astrocytes and promote vascular regeneration, thus reducing infarct size and facilitating neural recovery in rats (Hong et al., 2020). rTMS can promote upregulation of synaptic plasticity related protein expression, which is beneficial for the remodeling of synaptic ultrastructure (Qian et al., 2023). Additionally, rTMS can reduce mitochondrial damage, maintain mitochondrial membrane integrity, and mitigate oxidative stress damage to neurons (Zong et al., 2020). Although rTMS is widely used in the clinical management of stroke, its underlying mechanism remains unclear.

When an ischemic stroke occurs, cytotoxic edema leads to an increase in cell volume, thereby restricting the diffusion of water molecules within the cells. One of the characteristic pathophysiological features of ischemic stroke is the disruption of the blood–brain barrier (BBB). Following an ischemic stroke, increased permeability of the BBB and elevated expression of aquaporins in astrocytes can impair the glymphatic system and decrease cerebrospinal fluid flow, thereby exacerbating brain edema and impacting limb motor function (Ji et al., 2021; Zhu et al., 2024). Animal studies have shown that reduced cerebrospinal fluid (CSF) interstitial fluid (ISF) clearance severely damages the glymphatic system (Mestre et al., 2020). Early inflammatory responses protect neural tissue by clearing dead cell debris or harmful substances; however, excessive neuroinflammation is associated with BBB disruption and more severe neurological damage (Candelario-Jalil, Dijkhuizen & Magnus, 2022). Inflammation has a dual role in ischemic injury: in the early stages of an ischemic stroke, it contributes to brain damage, but in later stages, it promotes neural recovery by facilitating neurogenesis, angiogenesis, and neuronal plasticity (Candelario-Jalil, Dijkhuizen & Magnus, 2022). Therefore, reducing the inflammatory response and preventing the excessive release of inflammatory mediators is crucial for optimizing the microenvironment conducive to neuronal survival after a stroke (Qin et al., 2019). The DTI-ALPS index offers a non-invasive approach for researching the human glymphatic system. Qin et al. (2023) found that glymphatic system dysfunction is associated with subacute ischemic stroke. Toh, Siow & Castillo (2021) found that the DTI-ALPS index gradually increases with the time after stroke onset, potentially indicating the recovery of the glymphatic system after stroke injury. Pathological and physiological changes in the glymphatic system may play a crucial role in cerebral edema after stroke, but further basic research is needed to determine whether rTMS can target the glymphatic system as a therapeutic mechanism.

During the follow-up period, in the HF-rTMS group, the DTI-ALPS index of the non-stimulated hemisphere demonstrated a statistically significant decrease, while the DTI-ALPS index in the stimulated hemisphere showed no significant change. Notably, the decrease in the DTI-ALPS index was negatively correlated with the improvement of upper limb motor function in the non-stimulated hemisphere. These preliminary findings suggest that high-frequency rTMS, while facilitating motor recovery, could influence the glymphatic system. Given that the DTI-ALPS index is an indirect imaging surrogate for the glymphatic system, such reductions may not simply indicate “impaired clearance efficiency” but rather reflect a return from a post-stroke compensatory state toward homeostasis. A possible explanation is that high-frequency rTMS to the infarcted hemisphere M1 may modulate the activity of the non-stimulated hemisphere through transcallosal inhibition, thereby influencing neurovascular unit activity linked to glymphatic function (Iliff et al., 2013; Vucic et al., 2023). By restoring interhemispheric excitability balance, HF-rTMS may alleviate these pathological compensations, leading to a normalization of DTI-ALPS index in the non-stimulated hemisphere (intact side). In addition, ischemic stroke leads to AQP4 polarity disruption and perivascular astrocyte edema, which hinders the ability to regulate fluid transport (Mestre et al., 2018). Even though HF-rTMS enhanced neural excitability, it did not show any changes in DTI-ALPS in the stimulated hemisphere (infarct side).

In this study, longitudinal analysis showed that the DTI-ALPS index of the LH-rTMS group showed an increasing trend (although not statistically significant), but there was an improvement in upper limb motor function. This preliminary observation could potentially be attributed to two mechanistic pathways: (1) LF-rTMS might modulate interhemispheric connectivity by suppressing pathological hyperexcitability in the contralesional hemisphere (intact side), thereby attenuating its maladaptive inhibitory influence on the infarct side. (2) It may promote the clearance of metabolic waste by regulating blood–brain barrier (BBB) permeability, thereby reducing metabolic burden, manifested as a mild increase in DTI-ALPS (Vazana et al., 2020; Vejdani Afkham et al., 2022; Petrovskaya et al., 2023). Notably, in the low-frequency rTMS group, there were no significant changes in the DTI-ALPS index in either hemisphere, which might be due to the lesser impact of low-frequency stimulation on interhemispheric networks in the short term (Lefaucheur et al., 2020). rTMS at different frequencies may exert differential effects on glymphatic function, and this could have clinical implications regarding the choice of rehabilitation strategies. Future studies could integrate functional MRI and dynamic contrast-enhanced MRI to further elucidate the dynamic effects of rTMS on glymphatic flow.

This study has several limitations. First, the sample size is relatively small. Second, the short follow-up period of only two weeks makes it impossible to verify long-term effects. Third, the duration of cerebral infarction varies among subjects. The different stages of the disease are crucial factors influencing the treatment strategy when using TMS. Previous studies have indicated that enhancing cortical excitability in the infarcted hemisphere during the acute and subacute phases of stroke is a key strategy for improving upper limb neurological function in patients (Du et al., 2019). In the subacute and chronic phases of stroke, reducing excitability in the non-stimulated hemisphere might be more effective in promoting interhemispheric inhibitory homeostasis (Hsu et al., 2012; Chen et al., 2022). In the future, it is necessary to combine longitudinal studies with multimodal data to elucidate the potential value of the DTI-ALPS index as an intervention target and explore its specific manifestations at different stroke stages.

## Conclusions

This study preliminarily suggests that high-frequency repetitive transcranial magnetic stimulation (HF-rTMS) may modulate glymphatic system function, as evidenced by a reduced DTI-ALPS index in the non-stimulated hemisphere, which inversely correlates with upper limb motor recovery in ischemic stroke (IS) patients. Conversely, low-frequency rTMS (LF-rTMS) improved motor function without significant alterations in DTI-ALPS index, implying distinct mechanistic pathways between frequency-specific interventions. These findings highlight the potential of DTI-ALPS as a biomarker for evaluating rTMS-induced neurovascular remodeling. However, due to the short duration and limited sample size of the longitudinal data, further investigation is needed to validate these observations.

##  Supplemental Information

10.7717/peerj.20709/supp-1 Supplemental Information 1 Longitudinal changes in UL motor function of participants in the low-frequency rTMS group and high-frequency rTMS group

10.7717/peerj.20709/supp-2 Supplemental Information 2 Raw data

10.7717/peerj.20709/supp-3 Supplemental Information 3 Protocol

10.7717/peerj.20709/supp-4 Supplemental Information 4 CONSORT list

10.7717/peerj.20709/supp-5 Supplemental Information 5 CONSORT flow diagram

## Additional Information and Declarations

### Data Availability

The following information was supplied regarding data availability: The raw data are available in the Supplementary File.

### Clinical Trial Registration

The following information was supplied regarding Clinical Trial registration: ChiCTR2100047637.
