# Supplementary material for: Repetitive transcranial magnetic stimulation promotes the recovery of upper limb motor dysfunction in ischemic stroke patients: a DTI-based glymphatic system imaging prospective study
Source: PeerJ. 2026 Feb 3;14:e20709. doi: 10.7717/peerj.20709 (PMC12880090; doi:10.7717/peerj.20709)
Supplement: Supplemental Information 1 [file peerj-14-20709-s001.docx]

**table_s1 Longitudinal Changes of clinical characteristics**

| **Wilcoxon signed rank test** | | | |
| --- | --- | --- | --- |
|  | Variable | P-value | P-value (FDR) |
| LF-rTMS | NIHSS | **0.019** | **0.046** |
|  | Brunstrom | **0.008** | **0.025** |
|  | Fugl-Meyer Assessment | **0.000** | **0.000** |
|  | Motor assessment scale | 0.059 | 0.101 |
|  | Modified ashworth scale | 0.206 | 0.225 |
|  | Wolf Motor Function Test | **0.003** | **0.012** |
|  | SWMT | 0.059 | 0.101 |
|  | ADL | **0.000** | **0.002** |
|  | QOL | 0.144 | 0.192 |
| HF-rTMS | NIHSS | **0.002** | **0.008** |
|  | Brunstrom | **0.007** | **0.018** |
|  | Fugl-Meyer Assessment | **0.000** | **0.005** |
|  | Motor assessment scale | 0.157 | 0.261 |
|  | Modified ashworth scale | 1.000 | 1.000 |
|  | Wolf Motor Function Test | **0.007** | **0.018** |
|  | SWMT | 0.196 | 0.261 |
|  | ADL | **0.001** | **0.006** |
|  | QOL | 0.174 | 0.261 |

Computed by using Wilcoxon signed rank test. * indicates P < 0.05; **indicates P < 0.01; ***indicates P < 0.001.

NIHSS, National Institutes of Health Stroke Scale; WMFT, Wolf Motor Function Test; ADL, activities of daily living; QOL, quality of life.
