# Supplementary material for: Repetitive transcranial magnetic stimulation promotes the recovery of upper limb motor dysfunction in ischemic stroke patients: a DTI-based glymphatic system imaging prospective study
Source: PeerJ. 2026 Feb 3;14:e20709. doi: 10.7717/peerj.20709 (PMC12880090; doi:10.7717/peerj.20709)
Supplement: Supplemental Information 3 [file peerj-14-20709-s003.docx]

**The Impact of Different rTMS Protocols on the Rehabilitation of Hand Function in Subacute Stroke Patients**

**Experimental Design**

A multicenter, prospective, randomized, controlled, single-blind trial.

**Experimental Subjects**

Patients with hemiplegia due to stroke admitted to the Department of Rehabilitation Medicine at the First Affiliated Hospital of University of South China, included in the study according to the inclusion and exclusion criteria.

**Inclusion criteria**

(1) Initial stroke episode, consistent with the 2021 American Heart Association/American Stroke Association diagnostic criteria for stroke.

(2) Unilateral presentation characterized by persistent upper limb motor impairment. Participants must exhibit hand motor function within Brunstrom stages II to V and have a modified Ashworth scale score not exceeding Grade III. It was required that upper limb motor function was normal prior to the stroke.

(3) Disease duration of more than 14 days but less than six months post-stroke.

(4) Right-handed, age range of 18 to 80 years.

**Exclusion criteria**

(1) Pain and functional limitations in the affected upper limb caused by other etiologies.

(2) Recurrent stroke or other disease progression.

(3) Individuals with TMS contraindications such as metal, electronic device implants, and skull defects in the body.

(4) Has a personal or family history of epilepsy, or is currently taking medication that can alter cortical excitability.

(5) Individuals with severe mental abnormalities, cognitive, speech, and hearing impairments who are unable to cooperate with examinations and treatments.

(6) Combined with severe heart and lung dysfunction and other important organ dysfunction.

(7) Pregnancy.

(8) Participate in other clinical trials that affect the evaluation of the results of this study.

**Withdrawal Criteria**

(1) Any medical condition during the study that the researcher considers the subject should not continue from a medical perspective.

(2) The patient or their family requests to terminate the trial.

(3) Low compliance, not following the prescribed treatment, or incomplete data affecting the evaluation of efficacy.

**Baseline Data Collection**

All groups collect baseline data before rTMS treatment.

(1) Demographic information: gender, age, body mass index, right-handedness, etc.

(2) Medical history: disease course, lesion location, hemiplegic side, carotid artery stenosis or occlusion, NIHSS score, past history (hypertension, diabetes, hyperlipidemia), smoking history, alcohol abuse history, etc.

(3) Auxiliary examination: fibrinogen, blood lipids, plasma homocysteine, head CT/MRI, carotid ultrasound, CTA or MRA, etc.

(4) Hand function assessment: Fugl-Meyer score, MAS score, Modified Ashworth Scale, hand grip/pinch strength assessment, hand sensation assessment, finger dexterity assessment, etc.

(5) Electrophysiological examination indicators: record motor evoked potentials (MEPs) from both cerebral hemispheres, record the latency and amplitude of MEPs, motor threshold rMT, calculate central conduction time.

(6) Biochemical indicators: oxidative stress indicators: serum malondialdehyde (MDA), superoxide dismutase (SOD), endothelin-1 (ET-1); metabolic indicators; serum brain-derived neurotrophic factor (BDNF) levels.

(7) Functional MRI assessment of cortical neural networks.

**Intervention Measures (Intervention Plans for Experimental and Control Groups)**

All subjects undergo 2 weeks of standardized basic treatment and repetitive transcranial magnetic stimulation treatment.

(1) Basic treatment: All included subjects receive routine drug treatment and comprehensive rehabilitation treatment.

(2) Repetitive transcranial magnetic stimulation treatment plan: All experimental equipment is the magnetic field stimulator (YRD CCY-1) from Wuhan Yiruid Company, 8-shaped coil. LF-rTMS group: Low-frequency (100% rMT, 1 Hz, 1200 pulses) on non-infarcted hemisphere. high-frequency rTMS (100% rMT, 10 Hz stimulation, 1200 pulses) on the infarcted hemisphere.

**Allocation of intervention measures**

Randomization scheme, Blinding method, Concealment

**Outcome Measures (Efficacy Indicators, Safety Indicators)**

(1) Primary endpoint indicators: Fugl-Meyer upper limb motor function score at the end of rTMS intervention, and 1 month, 3 months, 6 months after intervention, and the change compared to baseline.

(2) Secondary endpoint indicators:

MAS score, Modified Ashworth Scale, hand grip/pinch strength assessment, hand sensation assessment, finger dexterity assessment at the end of rTMS intervention, and 1 month, 3 months, 6 months after intervention, and the change compared to baseline.

Assessment of cortical function (MEP amplitude, latency, CMCT, functional MRI) at the end of rTMS intervention, and 1 month, 3 months, 6 months after intervention, and the change compared to baseline.

NIHSS score at the end of rTMS intervention, and the change compared to baseline.

Assessment of daily living ability and long-term quality of life 6 months after the end of rTMS intervention.

(3) Safety assessment:

Record whether adverse events occurred during the visit period, record the number and proportion of adverse events, and truthfully fill in the adverse event report form, detailing the specific manifestations, severity, and relationship with this trial.
