# Supplementary material for: Repetitive transcranial magnetic stimulation promotes the recovery of upper limb motor dysfunction in ischemic stroke patients: a DTI-based glymphatic system imaging prospective study
Source: PeerJ. 2026 Feb 3;14:e20709. doi: 10.7717/peerj.20709 (PMC12880090; doi:10.7717/peerj.20709)
Supplement: Supplemental Information 5 [file peerj-14-20709-s005.docx]

**Assessed for eligibility (n=60)**

**Excluded (n=6)**

insufficient MRI (n=4)

multiple infarctions in both hemispheres (n=1)

abnormal blood lymphocytes (n=1)

1

¨  Other reasons (n= )

**Analysed (n = 17 )**

**Lost to follow-up (subject gives up) (n=10)**

**Allocated to high-frequency rTMS group (n=27)**

**Lost to follow-up (subject gives up) (n=8)**

**Allocated to low-frequency rTMS group (n=27)**

**Analysed (n = 18)**

**Excluded from analysis (insufficient MRI) (n= 1)**

**Allocation**

**Analysis**

**Follow-Up**

**Randomized (n=54 )**

**Enrollment**

.

Figure 1. CONSORT 2010 flow diagram (adapted from <http://www.consort-statement.org/>).
